# Survival and Prognostic Factors in Mixed Cryoglobulinemia: Data from 246 Cases

**DOI:** 10.3390/diseases6020035

**Published:** 2018-05-03

**Authors:** Cesare Mazzaro, Luigino Dal Maso, Endri Mauro, Valter Gattei, Michela Ghersetti, Pietro Bulian, Giulia Moratelli, Gabriele Grassi, Francesca Zorat, Gabriele Pozzato

**Affiliations:** 1 Clinical and Experimental Onco-Haematology Unit, CRO Aviano National Cancer Institute IRCCS, 33081 Aviano, Italy; cesare.mazzaro@gmail.com (C.M.); vgattei@cro.it (V.G.); pbulian@cro.it (P.B.); 2 Epidemiology and Statistic Unit, CRO Aviano National Cancer Institute IRCCS, 33081 Aviano, Italy; dalmaso@cro.it (L.D.M.); michela.ghersetti@aas5.sanita.fvg.it (M.G.); 3 Department of Internal Medicine, Pordenone General Hospital, 33170 Pordenone, Italy; endri76@libero.it; 4 Department of Clinical and Surgical Sciences, University of Trieste, 34121 Trieste, Italy; giulia.moratelli@gmail.com (G.M.); francesca.zorat@asuits.sanita.fvg.it (F.Z.); 5 Department of Life Sciences, University of Trieste, 34121 Trieste, Italy; ggrassi@units.it

**Keywords:** hepatitis C virus, mixed cryoglobulinemia, interferon alpha, steroids, rituximab

## Abstract

Introduction: The clinical and therapeutic management of mixed cryoglobulinemia (MC) remains a subject of controversy. In addition, most studies have not recorded the long-term follow-up and the outcome of these cases. Material and Methods: We enrolled 246 patients affected by MC who were consecutively admitted to our Department from January 1993 to February 2013. Clinical and biological data had been recorded until June 2014. Results: The median age (at diagnosis) was 60 years (range 26–83). The aetiology was HCV in 95% of patients, HBV in 3% and “essential” in 2%. HCV genotype was 1b in 57%, genotypes 2–3 in 43%. MC was Type II in 203 of the cases (87%) and Type III in 52 (13%). The most frequent clinical manifestations were purpura (72%), chronic liver disease (70%), glomerulonephritis (35%), arthralgias (58%), peripheral neuropathy (21%), non-Hodgkin lymphoma (15%) and cutaneous ulcers (3%). Purpura, arthralgias, peripheral neuropathy, glomerulonephritis and non-Hodgkin lymphoma were more frequently observed in Type II than in Type III MC (*p <* 0.05). Treatments were interferon (IFN) or Pegilated-IFN (PEG-IFN) alone or plus Ribavirin (RIBA) in 101 cases, steroids with or without alkylating agents in 33 cases, Rituximab in 8 patients. The complete clinical, virological and immunological responses were associated with PEG-IFN plus RIBA. Severe infections were associated with renal failure. At 10 years, the overall survival rate was 71% in Type II MC and 84% in Type III (*p <* 0.053). Conclusions: From our data, antiviral therapy is the first-line therapy in HCV-related MC, whereas steroids, alkylating agents and Rituximab should be considered as a second-line therapy. Given the heterogeneity of the disease, the role of these different therapeutic strategies should be checked in randomized controlled trials.

## 1. Introduction

Cryoglobulinemia is a rare disease characterized by the presence in the serum of immunoglobulins able to precipitate with cold temperature and to re-dissolve with rewarming. According to Brouet, the cryoglobulinemias are classified in three types: in Type I the cryoglobulins are formed by monoclonal immunoglobulins only, commonly IgMs—this type of cryoglobulin is confined to cases of Waldenström’s disease or other similar lymphoproliferative disorders. In Type II and Type III (so-called mixed cryoglobulinemias) (MC) the cryoglobulins are immune-complexes composed of polyclonal IgGs, antigen(s) and monoclonal or polyclonal IgMs respectively. The IgM are endowed with rheumatoid factor activity, that is, against polyclonal IgG [1,2,3]. Type II and Type III are associated with connective tissue diseases, lymphoproliferative disorders and several chronic infections.

In the past, when MC was not associated with a well-defined disease, the syndrome was designed as “essential”. After the discovery of the hepatitis C virus (HCV) in 1989, it has become clear that more than 90% of the MC cases carried the HCV [4,5]. 

The clinical manifestations of MC are secondary to the deposition of immune-complexes in several organs and tissues. MC may determine not only purpura, arthralgias and asthenia but also serious skin lesions (large ulcers) and neurologic and renal involvement [6,7]. Since almost the totality of the patients are carriers of an HCV infection, antiviral therapy is the current standard of care for patients with mild to moderate disease, whereas more aggressive treatments, like corticosteroids or plasmapheresis, are needed in the patients showing severe organ involvement (skin ulcers, peripheral neuropathy, glomerulonephritis). In these cases, several authors evaluated the efficacy and safety of anti-CD20 monoclonal antibodies treatment [8,9]). 

Given the great heterogeneity of this disease, showing several pathological conditions and multi-organ involvement in the same patient, the long-term follow-up and the outcome of these cases is not clear, since often several specialists follow them separately. In addition, clear indications for therapy, shared by most clinicians, are lacking. No well-defined rules are established for MC concerning when the treatment should be initiated, or regarding what the endpoint of the therapy should be. In fact, some patients do not show any hepatic damage but have renal or neurological involvement, while others show severe hepatic disease or autoimmune disorders, therefore, they have the major contraindications to the former standard antiviral therapy (interferon and ribavirin). Since our Hospital follows all cases affected by MC in a single centre for this purpose, we have had the opportunity to retrospectively evaluate the long-term outcome of a large group of MC patients. Here we report also the clinical data on the efficacy and the safety of several treatments of 246 cases of MC followed since 1997.

## 2. Patients and Methods

Two hundred forty-six consecutive patients (M/F ratio 1.2) affected by MC were included in this study. The median age at the onset was 60 years (range 26–83). All cases were enrolled between January 1993 and February 2013 in the Department of Internal Medicine of the Pordenone General Hospital. The inclusion criteria of this retrospective study included the presence of the usual clinical symptoms (i.e., purpura, asthenia, arthargias) associated with dose levels of cryoglobulins (cryocrit > 1% on at least 2 occasions). We excluded only the cases infected with HIV. A fraction of the cases diagnosed in the same department between 1985 and 1993 (*n* = 98) were also retrospectively retrieved.

Clinical and biological data were recorded for each patient at onset and at each visit, scheduled every 3 months (apart from the antiviral therapy period) and information was collected until June 2014. Overall survival (OS) was calculated for all patients from the time of diagnosis until death by any cause or last follow-up.

In addition to the common liver, kidney and haematological parameters, the laboratory assessment included determination of complement components, rheumatoid factor and cryoglobulin serum levels. MC was defined as Type II when polyclonal IgG and monoclonal IgM immunoglobulins, endowed with rheumatoid factor activity, formed the immuno-complexes. Mixed cryoglobulinemia was classified as Type III and Type I when the immuno-complexes were formed by polyclonal or monoclonal immunoglobulin, respectively.

All the patients with renal involvement underwent kidney biopsy. Liver biopsy was performed only in the patients with biological signs of chronic liver disease. The disease activity and fibrosis were assessed according to METAVIR [10]. The diagnosis of B-cell non-Hodgkin lymphoma (B-NHL) was done according to the World Health Organization’s classification [11]. We recorded all the treatment features.

**Skin lesions**: The severity of the skin involvement was determined as follows: a score of 0 indicated the absence of skin lesions. A score of 1, the presence of less than 10 purpura spots on the lower legs. A score of 2, the presence of more than 10 spots on the lower legs. A score of 3, the extension of the spots to the upper leg and/or the abdomen and a score of 4, the presence of skin ulcers and/or gangrene.

**Arthralgias**: To assess the severity of the arthralgias a clinical score was used: 0 indicated no arthralgias, 1 for occasional arthralgias, 2 for continuous arthralgias, 3 for intense arthralgias with impairment of movement.

**Response to therapy**: To assess the efficacy of the treatments, we split the response into four separate categories [12]: (1) Virological response, (2) Biochemical response, (3) Immune response, (4) Clinical response.

(1) Virological response: effect of treatment on HCV-RNA. Sustained virological response (SVR): loss of HCV-RNA at the end of follow-up. Relapse: loss of HCV-RNA at the end of treatment but reappearance of viral replication during follow-up. No response: persistent HCV-RNA positivity during therapy and follow-up.
(2) Biochemical response: effect of therapy on ALT, normal value was considered 40 IU/L. Complete responses: normalization of the serum ALT level during treatment followed by normal ALT values lasting for 6 months after discontinuation of therapy. No response: ALT out of normal value during treatment and follow-up. Relapse: normalization of the serum ALT level during treatment followed by return to abnormal values during follow-up. In some patients, this parameter was not considered, since the ALT level was normal at the beginning of the treatment.
(3) Immune response: effect of therapy on serum RF concentration and on cryocrit level. Complete response: normalization of serum RF concentration and disappearance of circulating cryoglobulins. Partial response: reduction (but not normalization) of RF and cryoglobulins >50%. No response: Reduction <50% of RF and cryocrit levels or stable levels. Relapse: partial or complete normalization of serum RF and cryoglobulins during therapy followed by return to higher values during follow-up.
(4) Clinical response: effect of therapy on the clinical manifestations of the disease (including purpura, arthralgia and weakness). Complete response: disappearance of all clinical signs of the disease. Partial response: improvement of the clinical symptoms (reduction of the purpura score >50%). No response: reduction of the purpura score <50% or stable disease. Relapse: partial or complete normalization of clinical symptoms during therapy followed by return to higher score after the end of treatment.

In patients with renal involvement, the complete response was defined as the return to the normal level of the serum creatinine associated with the disappearance of proteinuria. A partial response was defined as the decrease of creatinine and proteinuria by more than 50% and a minimal response as the decrease of creatinine and of proteinuria by less than 50%. The decrease of proteinuria by less than 10% was considered as no response.

In the cases with peripheral neuropathy, the response was defined as an improvement of the electromyography at two successive evaluations.

## 3. Statistical Analysis

Descriptive statistics included the median age, range and interquartile range (IQR) as a measure of variability. Univariate analysis included the Fisher exact test as appropriate to compare categorical variables and the nonparametric Log-rank test to compare continuous variables. The 5-year and 10-year OS was computed by the Kaplan-Meier method and the Log-rank test was used to test the differences between subgroups. Differences were considered significant when *p*-values were <0.05. The study was performed in accordance with the ethical standards of the Helsinki Declaration and it was approved by the institutional review board.

## 4. Results

### 4.1. Patient Characteristics

The clinical features of the 246 patients with MC are shown in Table 1. The median age at diagnosis was 60 years (range: 26–83) and 160 patients were female (65%). Clinical manifestations included skin involvement (purpura) in 177 patients (72%), arthralgias in 143 patients (58%), peripheral neuropathy in 52 patients (21%). Liver involvement was found in 217 patients (88%) including chronic hepatitis in 173 patients and cirrhosis in 44 patients (8 of them with hepatocellular carcinoma), renal involvement in 19 patients (8%), B-NHL in 38 patients (15%). The median cryocrit level was 3.0%. At the onset, anti-HCV antibodies were found in 243 patients (99%) while the HCV-RNA was detectable in 235 patients (95%). The HCV genotype was available in 150 patients: genotype 1–4 in 116 patients (77%), genotype 2–3 in 34 patients (23%). There were 11 patients (4.5%) found positive for HBsAg. Among these cases, 3 were positive for both HBV-DNA and HCV-RNA, while 8 were positive for HBV-DNA only (3.2%). Only 3 patients (1.2%) were negative for both HBV and HCV, and, therefore, these cases were considered as carrier of the “essential” MC (1 Type II and 2 Type III).

Comparing the two types of MC, the patients with Type II had more severe purpura (*p <* 0.01) and arthalrgias (*p <* 0.04), they also were affected more frequently with peripheral neuropathy (*p <* 0.03) and B-cell NHL (*p <* 0.01). From the biochemical point of view, the patients with Type II had higher cryoglobulin (*p <* 0.05) and rheumatoid factor levels (*p <* 0.03) and lower complement component 4 serum levels (*p <* 0.04). 

The renal biopsy was performed in the 17 patients showing renal involvement, in these cases the histology showed the presence of a membranoproliferative glomerulonephritis Type I in 16 cases, whereas 1 had a mesangial proliferative glomerulonephritis (Table 1). Among the patents with renal involvement, only 2 (12%) were carriers of Type III MC.

In the 38 patients with NHL, Type II MC was the most prevalent (35 cases, 92%). The most frequent histological subtype of NHL was marginal zone lymphoma (8 nodal marginal zone, 15 splenic marginal zones, 6 non-nodal marginal zones and 3 mucosa-associated marginal zone lymphomas). Also found were 5 lymphocytic lymphomas and 1 diffuse large B-cell lymphoma. 

### 4.2. Follow-Up of the Patients

**Figure 1:**
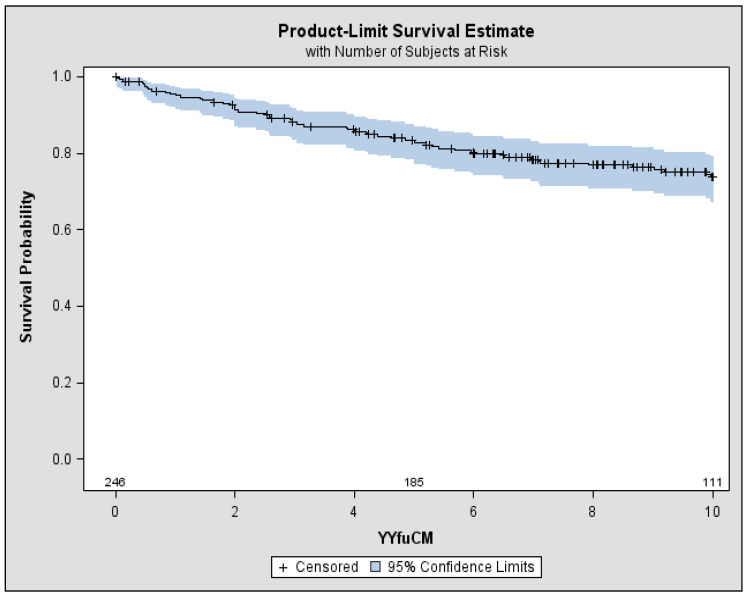
Overall survival of the patients affected by HCV-positive MC. 5-year Survival 84% (95% CI: 78–88%), 10-year Survival 74% (95% CI: 67–79%) MC: mixed cryoglobulinemia, CI: confidence intervals.

**Figure 2:**
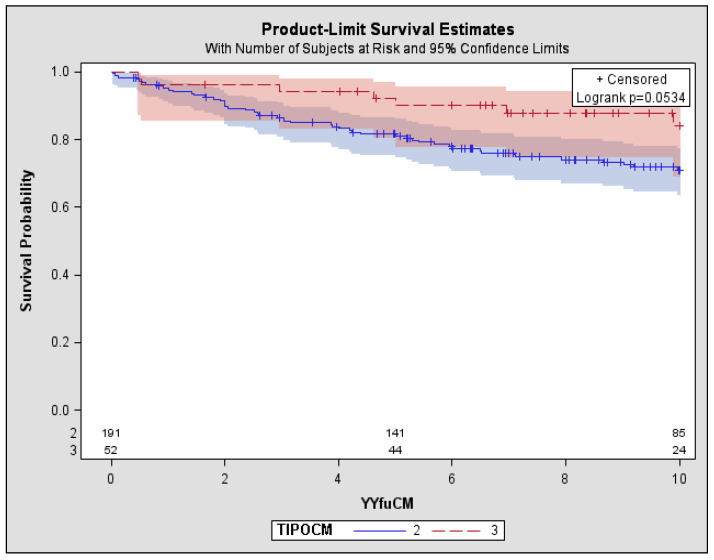
Observed 10-year overall survival in MC Type (II vs. III). Type II: 5-year Survival 81% (95% CI: 75–86%) 10-year Survival 71% (95% CI: 63–77%). Type III: 5-year Survival 92% (95% CI: 80–97%) 10-year Survival 84% (95% CI: 69–92%). MC: mixed cryoglobulinemia, CI: confidence intervals.

After a median follow-up of 9.2 years, 72 patients (29%) had died: 63 with Type II MC and 9 with Type III MC (Table 1 and Figure 1). In the carriers of Type II MC, 32 patients died due to cirrhosis complications (51%): hepatic encephalopathy, oesophageal varices bleeding or liver failure. Out of the remaining 31 patients: 10 died due to heart failure (16%), 6 to kidney failure (10%), 8 to severe infections including septicaemia and pneumonia, 3 to intestinal vasculitis, 1 to myocardial infarction, 3 for NHL. Among the 9 patients with Type III CM, 6 cases (67%) died due to cirrhosis complications, 1 due to heart failure and 2 to kidney failure (both in haemodialysis) (Table 2). Overall survival rate at 10 years was 74%, with a significant difference (*p <* 0.05) between Type III MC (84%) and Type II MC (71%) (Figure 2).

### 4.3. Effects of Therapeutic Regimens

One hundred ninety patients (77%) underwent antiviral and/or immunosuppressive therapy. The antiviral regimens were interferon-alpha (IFN) as monotherapy in 61 cases, IFN plus ribavirin (RIBA) in 20 cases, pegylated interferon-alpha (PEG-IFN) plus RIBA in 21 cases and direct antiviral agents (DAAs) in 19 cases. The immunosuppressive agents were corticosteroid alone in 52 cases, alkylating agents plus corticosteroid in 8 cases and rituximab in 6 cases. A small fraction of patients (12 cases) underwent plasmapheresis (Table 3). During follow-up, symptomatic treatments (FANS, diuretic, ACE-inhibitors, beta-blockers) were used in 66 cases. It was possible to retrospectively evaluate the efficacy, the safety, the side effects and the outcome of these different treatments.

IFN as monotherapy provided sustained virological as well as complete clinical, immunological and biochemical response in 15 patients (24%). Combination therapy (IFN and RIBA or PEG-IFN and RIBA) provided a complete clinical, virological, biochemical and immunological response in 15 patients (36%). In the patients treated with steroids (as monotherapy or in combination with alkylating agents), we did not observe any virological response, while a good control of the symptoms (clinical response) was obtained in a large fraction of cases. In this group, a small fraction of patients showed a biochemical response and a partial immunological response. Among the 21 cases affected by glomerulonephritis, a complete renal response was obtained in 8 cases (38%) after antiviral therapy. There were 3 cases with severe vasculitis, non-responders to IFN and RIBA, were treated subsequently with plasmapheresis, steroids and/or Rituximab. These 3 patients obtained the complete renal response, clinical and partial immunological responses but not the virological response. Even the 19 cases affected by low-grade B-NHL were treated with antiviral therapy. A complete response was achieved by 7 of them (37%): 2 cases with IFN as monotherapy, 1 case with IFN and RIBA, 2 cases with PEG-IFN and RIBA. Two cases were re-treated with DAAs without haematological response. Altogether, 112 cases were treated with antiviral therapy. During this treatment, 3 patients (3%) developed a severe infection. In the long-term follow-up 14 patients died (14%), most of them for the progression of the liver disease. Nineteen patients (10 treatment-naive and 9 previously treated with PEG-IFN) received sofosbuvir-based regimen or other DAAs, individually tailored according to the latest guidelines. As of December 2016, the median length of follow-up of these cases was 17 months (range 13–21). After a four-week DAA therapy, all patients became HCV-negative and after 48 weeks, all cases obtained SVR. Conversely, sustained regression of purpura and arthralgias was observed in 8 cases (42%). Two cases with indolent marginal zone lymphomas did not show any haematological response: size and number of the involved nodes remained unchanged. In addition, the monoclonal B-cell population found in the peripheral blood in 4 cases did not disappear after recovery from HCV-RNA. 

Altogether, 72 cases were treated with corticosteroids (alone or in combination with alkylating agents or plasmapheresis). During these treatments, 5 patients (7%) developed severe infections. As expected, none obtained the virological response but 8 patients, refractory to antiviral therapy, obtained complete clinical response and a partial immunological response. In this group, 32 patients died (44%), the causes of death were not only severe infection but also liver, kidney or heart insufficiency, hepatocellular carcinoma, NHL and vasculitis. 

Six cases (all affected by Type II MC) were treated with Rituximab. Of these, 4 were carriers of MPGN and 2 of NHL. Rituximab provided a complete renal response in 1 case and complete immunologic and clinical response in 4 cases. One of the 2 cases affected by indolent B-cell NHL obtained a haematological partial response. Among the patients treated with Rituximab, 2 died (34%), 1 for NHL and 1, several years later, for severe infection.

**Figure 3:**
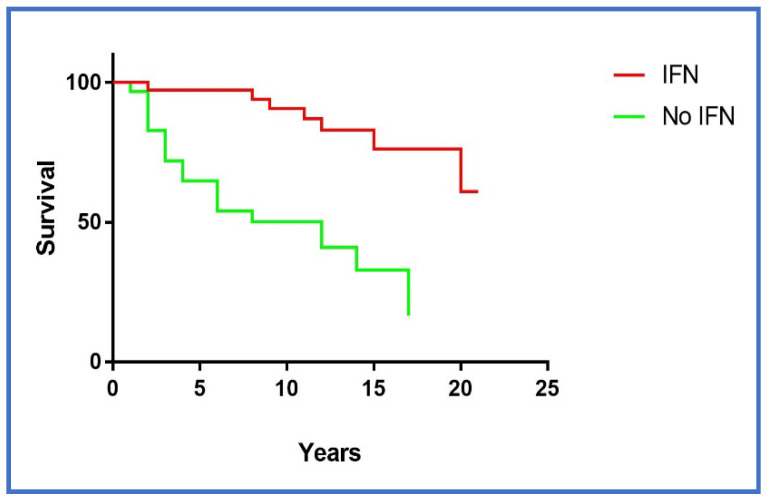
Survival rate according to the therapy. “IFN” includes patients who underwent interferon therapy (IFN) as monotherapy, IFN in combination with ribavirin (IFN + RIBA) and Pegilated IFN plus RIBA (PEG-IFN + RIBA) without a sustained virological response (non-responders or relapsers). “No IFN” includes patients who underwent immuno-suppressive treatments that is, steroids in combination with alkylating agents/rituximab and/or cyclosporine and/or cyclophosphamide. IFN vs. No IFN *p* < 0.0001.

Excluding the 49 patients who obtained the complete virological response, the survival of the cases treated with antiviral therapy (non-responders or relapsers) was significantly better (*p <* 0.0001) (Figure 3) than the survival of the patients who underwent only immuno-suppressive therapy (including Rituximab).

## 5. Discussion

This study describes the spectrum of clinical presentations and the causative factors of infective MC. Demographic features were in accordance with the previously reported series of HCV positive or negative patients [13,14,15,16]. The patients with Type II MC show higher cryoglobulin serum levels, lower complement component four than Type III MC and, clinically, have more frequently purpura, peripheral neuropathy, renal involvement and lymphoproliferative disorders. The more severe clinical impact of Type II MC seems to be correlated with the disease itself since the other clinical factors (age, gender, alcohol consumption, co-morbidities, etc.) and biological features (HCV genotype, HBV coinfection, severity of the chronic liver disease) are distributed similarly between Type II and Type III MC. 

To improve management of MC, the main goal of this study was the evaluation of the different therapeutic regimens providing interesting findings on efficacy and safety. All antiviral regimens based on interferon showed better efficacy in first-line therapy rather than in relapsed and/or refractory disease. The eradication of the HCV infection was achieved in over one-third of treated cases and it was associated with the clinical and immunological response. The finding of a better survival of the cases treated with antiviral therapy (even of non-responders or relapsed) as compared with those who underwent only immuno-suppressive treatments, does not mean that this therapy is useful independently of its outcome. In fact, the traditional antiviral therapy, based on the administration of alfa-IFN alone or in combination with RIBA, was endowed with several severe side effects, therefore, this treatment was not recommended for older patients, in uncompensated cirrhosis and in presence of cardiovascular diseases. In addition, there were several contraindications to the interferon therapy such as epilepsy, autoimmune diseases and skin diseases. As a consequence, the better outcome of the cases treated with antiviral therapy indicates that these patients showed less co-morbidity, had a less severe liver disease and, finally, were younger than untreated patients (57 ± 9 vs. 62 ± 7 *p <* 0.0001). 

As first-line therapy, the efficacy of corticosteroids alone was similar to corticosteroids with alkylating agents. However, there was a high rate of side effects of steroids and/or alkylating agents, especially severe infections. In the patients with large skin ulcers, severe glomerulonephritis or with hyper-viscosity syndrome, plasmapheresis in combination with corticosteroids showed good efficacy in about half the cases. In the relapsed and/or refractory disease with organ involvement (mainly skin ulcers or nephropathy), the anti-CD20 administration provided a better efficacy as compared to steroids with or without alkylating agents and to steroids plus plasmapheresis. Both aminotransferases serum levels and serum HCV-RNA concentration increased in half the rituximab-treated cases but common laboratory tests together with clinical assessment did not show any deterioration of liver function. Among these patients, only one died due to severe infection some years later. Several papers including, at present, 279 patients [8], have confirmed the safety of rituximab. Given this good ratio between efficacy and side effects, rituximab could be considered the therapy of choice in relapsed/refractory cryoglobulinemic vasculitis. However, no virological response has been achieved in patients treated with corticosteroids, alkylating agents and rituximab, therefore, within a variable time, the clinical and laboratory signs of the disease relapsed. 

Concerning the renal involvement in MC, this seemed to be associated with a poor prognosis as previously suggested by several papers [3,17,18,19]. In a previous paper [15], the 119 HCV-positive MC enrolled cases were divided in two groups: one without kidney involvement (103 cases) and one with glomerulonephritis (16 cases). The survival rates at 5 years were 87% in the group without kidney involvement and 50% in the group with kidney involvement. Severe infections (pneumonia, septic complications) were the causes of death. Similarly, in this study we highlighted that fatal infections occurred in Type II MC with nephropathy. Moreover, only in Type II MC we found fatal diffuse (pulmonary or intestinal) vasculitis and a severe lymphoproliferative disease, while no patient with Type III MC died due to severe infections, vasculitis or lymphoma. Uncompensated cirrhosis, heart failure, advanced renal failure were the main causes of death in Type III MC. 

In conclusion, from the long-term follow-up of our patients, three major considerations can be made: (1) Clinical manifestations of the vasculitis (skin, kidney, nerves) are more frequently in Type II MC than in Type III, (2) Antiviral treatment is the therapy of choice and rituximab plays a key role in the relapsed/refractory disease, replacing the other immuno-suppressive regimens, (3) Despite the heterogeneity of the clinical and laboratory aspects of the disease, Type II MC shows a poorer survival rate at 10 years than Type III MC. 

These results also indicate the need of a prompt antiviral therapy in MC, to avoid the late complications of the disease, even in absence of a liver involvement. Until now, the interferon-based antiviral therapy could be applied only in the initial phases of MC, in absence of autoantibodies or organ involvements. The impact of the new direct antiviral agents (DAA) including sofosbuvir, simeprevir, daclatasvir and ledipasvir (and others) on the long-term prognosis of MC is still poorly understood but these DAAs are able to eliminate the viral replication in nearly 100% of cases. In addition to having a high efficacy, these DAAs show a very low rate of side effects and can be used in any phase of the disease, even in presence of severe vasculitis, autoimmune disease or renal failure. As indicated by Artemova et al. [20] and also by Mazzaro and co-workers [21] in a recent paper, the DAAs show less efficacy on the clinical symptoms and on the immunological disorders associated with MC. In addition, the DDAs treatment was not able to eliminate the B-cell clonal population in peripheral blood. On the contrary, we [22] and other authors [23] had shown that the combination therapy (IFN or PEG-IFN and ribavirin) is effective in HCV-positive indolent NHL of different histologies (including lymphocytic lymphoma, marginal-zone lymphoma, lympho-plasmacytic lymphomas and even follicular lymphomas): in these cases, it is likely that the anti-proliferative properties of IFN played the leading role in the cure of lymphoproliferative diseases rather than its antiviral properties. In fact, some authors observed that a very prolonged (66 months) IFN therapy was able to counteract the severe immunological disorders induced by HCV infection, even after disappearing of serum HCV-RNA [24]. However, even in these recent papers [20,21] the follow-up was short and, as it occurs in the gastric mucosa after Helicobacter Pylori eradication, complete recovery from the lymphoproliferative disorder can be observed after months or years following the elimination of the infective agent.

## Figures and Tables

**Table 1:** Clinical, histological and biochemical features of the 246 patients affected by mixed cryoglobulinemia (MC).

Characteristics	All Cases (246)	Type II MC (191 Cases)	Type III MC (55 Cases)	*p* Type II vs. Type III
Male/Female ratio	0.53	0.52	0.57	ns
Mean age at the diagnosis (range)	60 (26–83)	60 (26–77)	60 (34–83)	ns
Mean follow-up (years) (range)	9.2 (1–27)	9.0 (1–10)	9.7 (0.5–10)	ns
Clinical features at presentation				
Purpura (%)	177 (72%)	148 (77%)	29 (53%)	<0.01
Arthralgias (%)	143 (58%)	120 (63%)	23 (42%)	<0.04
Sicca syndrome (%)	22 (9%)	21 (11%)	1 (2%)	ns
Raynaud phenomenon (%)	34 (14%)	29 (15%)	5 (9%)	ns
Skin ulcers (%)	7 (3%)	7 (4%)	0	ns
Peripheral neuropathy (%)	52 (21%)	47 (25%)	5 (9%)	<0.03
Chronic hepatitis (%)	173 (70%)	134 (70%)	39 (71%)	ns
Glomerulonephritis (%)	19 (8%)	17 (9%)	2 (4%)	ns
Non-Hodgkin’s lymphoma (%)	38 (15%)	35 (18%)	3 (5%)	<0.01
Biochemical and virological features				
Cryocrit (%)	3.8 ± 4.0	4.6 ± 4.6	2.0 ± 1.5	<0.05
Rheumatoid factor (U/L) NV < 40	296 ± 691	343 ± 748	157 ± 382	<0.03
C4 NV > 10	9.4 ± 7.0	9.0 ± 7.3	10 ± 4.5	<0.04
Anti-HCV antibodies	238			
HCV genotype 1–4	116 (47%)	93 (49%)	23 (42%)	ns
HCV genotype 2–3	34 (14%)	22 (12%)	12 (23%)	ns
HBsAg	11 (4.5%)	8 (5%)	3 (5%)	
Anti-HBs	22 (9%)	14 (7%)	6 (11%)	ns
AST U/L NV < 36	66 ± 64	68 ± 61	60 ± 37	ns
ALT U/L NV < 45	69 ± 58	71 ± 62	62 ± 38	ns
GGT U/L NV < 36	66 ± 23	68 ± 22	63 ± 24	ns
Creatinine NV < 1.0 mg%	1.0 ± 0.2	1.0 ± 0.2	1.0 ± 0.2	ns
Outcomes				
Death	72 (29%)	63 (33%)	9 (17%)	<0.05

C4: complement component four, HBsAg: hepatitis B surface antigen, anti-HBs: antibodies against hepatitis B surface antigen, AST: aspartate aminotransferase, ALT: alanino-aminotransferase, GGT: gammaglutamyl-transpeptidase.

**Table 2:** Causes of death in the 246 cases affected by mixed cryoglobulinemia.

Causes of Death	All Patients	Type II MC	Type III MC
Liver cirrhosis	38	32 (51%)	6 (67%)
Heart failure	11	10 (16%)	1 (11%)
Renal insufficiency	8	6 (10%)	2 (22%)
Severe infections	8	8 (13%)	0
Vasculitis	3	3 (5%)	0
B-cell lymphoma	3	3 (5%)	0
Myocardial infarction	1	1 (2%)	0
Total cases	72	63	9

MC: mixed cryoglobulinemia, Liver cirrhosis included all complications of the diseases (hepatic encephalopathy, oesophageal varices bleeding, liver failure, hepatocellular carcinoma).

**Table 3:** Efficacy and safety of different therapies in 180 cases of MC. As shown, the best virological responses have been obtained with DAA, while the best clinical and immunological responses have been with rituximab. Of note, some cases received more than one therapy (265 treatments for 180 cases).

Type of Therapy	All Patients	CM Type II	CM Type III	Clinical Response	Immunological Response	SVR	Refractory Disease	Severe Infection after Therapy
IFN as monotherapy (%)	61	50	11	15 (24%)	14 (22%)	15 (4%)	6 (9%)	2 (3%)
IFN+Ribavirine (%)	20	15	5	8 (40%)	4 (20%)	8 (40%)	2 (10%)	1 (5%)
Peg-IFN + Ribavirine (%)	21	18	3	7 (33%)	4 (19%)	7 (33%)	6 (28%)	0
Corticosteroids alone (%)	52	43	9	4 (7%)	3 (6%)	0	12 (23%)	2 (3%)
Alkylant + Corticosteroids (%)	8	8	0	3 (37%)	3 (37%)	0	3 (37%)	2 (25%)
Plasmaferesys + steroids (%)	12	11	1	4 (33%)	4 (33%)	0	1 (8%)	1 (8%)
Rituximab (%)	6	6	0	4 (66%)	4 (66%)	0	2 (33%)	1 (16%)
DAAs (%)	19	14	5	8 (42%)	6 (32%)	19 (100%)	1 (5%)	0
